# Online database of Power Hardware In-the-Loop tests

**DOI:** 10.1016/j.dib.2020.105128

**Published:** 2020-01-11

**Authors:** Eduardo García-Martínez, José Francisco Sanz, Jesús Muñoz-Cruzado, Juan Manuel Perié

**Affiliations:** a CIRCE - Research Centre for Energy Resources and Consumption, Parque Empresarial Dinamiza. Avda. Ranillas 3D, 1^a^ Planta, 50018, Zaragoza, Spain; b Instituto Universitario de Investigación CIRCE - (Universidad de Zaragoza - Fundación CIRCE), Edificio CIRCE, Campus Rio Ebro, C/ Mariano Esquillor Gómez, 15, 50018, Zaragoza, Spain

**Keywords:** Power Hardware in-the-Loop (PHIL), Online database, Digital real-time simulator (DRTS), Power amplifier (PA), Rapid prototyping, Smart grid test bed

## Abstract

The online database presented in this article provides information about Power Hardware In-the-Loop (PHIL) tests to allow the reproducibility of the experiments. The data were collected through published papers and manufacturer data sheets. The database is hosted on an open subversion platform, which allows a continuous improvement of the data. Furthermore, a GUI interface has been developed to ensure the integrity of the database and its traceability. The access to PHIL test data will facilitate reliable findings for the reproducibility of PHIL experiments.

**Table:** Specifications Table

Subject	Electrical and Electronic Engineering
Specific subject area	Power Hardware In-the-Loop, Rapid prototyping, Smart grid
Type of data	Microsoft Access® file
How data were acquired	Power Hardware In-the-Loop test literature System manufacturer data sheets
Data format	Raw and pre-processed Dynamic tables with GUI interface (.accdb)
Parameters for data collection	Power Hardware In-the-Loop test data, gathered in the scientific literature, and datasheet information of the systems.
Description of data collection	Published Power Hardware In-the-Loop (PHIL) tests have been reviewed, extracting the main information for reproducibility purposes. The resulting database was completed by gathering information from manufacturer data sheets of the components used in these tests.
Data source location	Research Centre for Energy Resources and Consumption, Parque Empresarial Dinamiza. Avda. Ranillas 3D, 1^a^ Planta, 50018 Zaragoza, Spain
Data accessibility	Public Repository The updated database file is made available in a GitHub repository Repository name: Zenodo Data identification number: 10.5281/zenodo.3571368 Direct URL to data: https://zenodo.org/record/3571368#.XfFJIPyCGUk

**Table:** 

**Value of the Data** • These data contain the main information gathered from Power Hardware In-the-Loop (PHIL) tests. The database can be used to reproduce PHIL tests and provide support in the selection process of PHIL systems. • Researchers can quickly find examples of PHIL experiments to obtain methods and insights to develop their own tests. • This database has been uploaded to an open subversion platform (GitHub) [1], where the scientific community has the potential to improve and update the data, giving a complete perspective of the current developed PHIL tests to date. • It is expected that this online database will be a useful tool which helps and boosts the improvement of the smart grid and the rapid prototyping of new systems.

## 1 Data

**Fig. 1:**
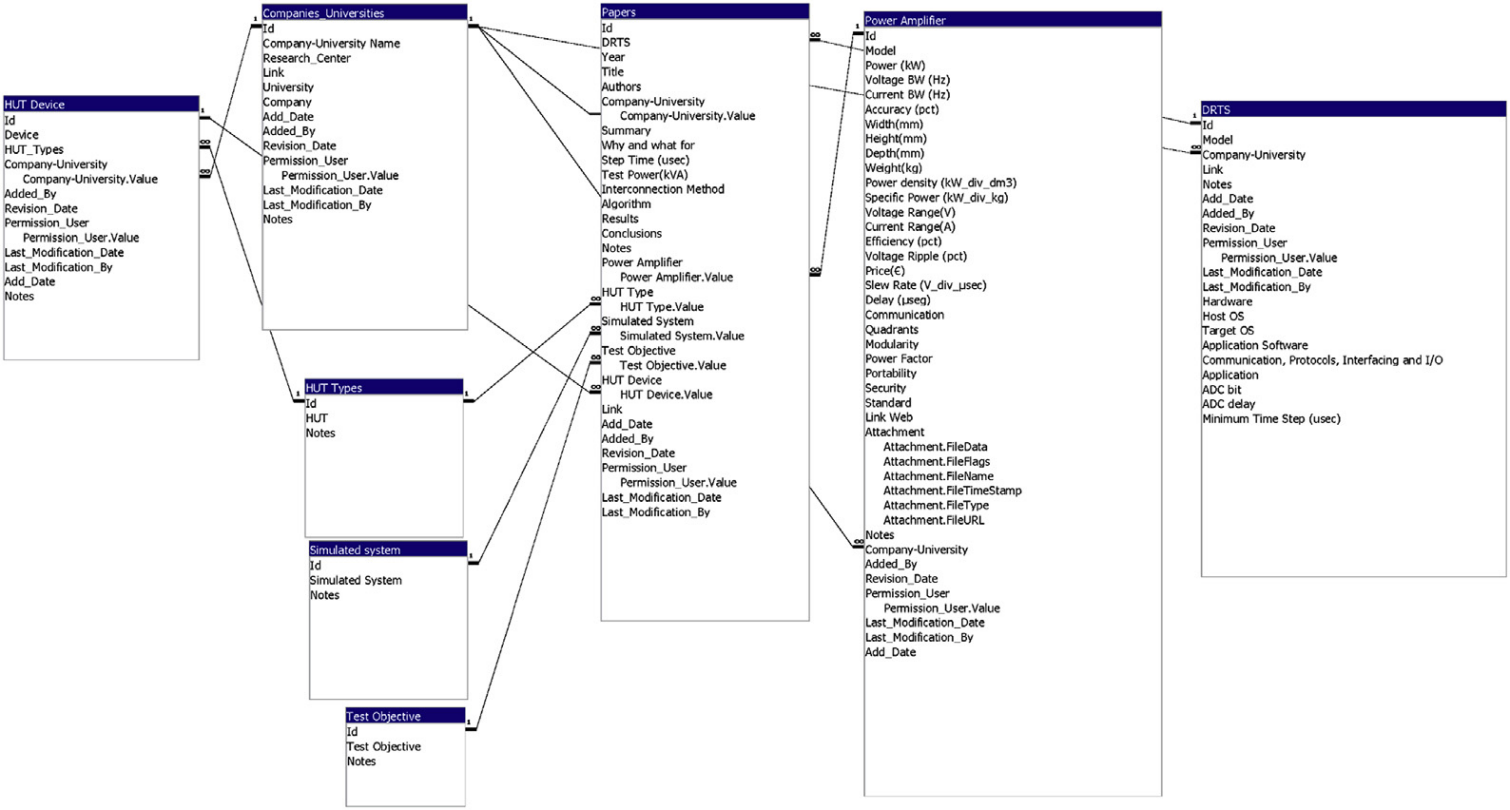
UML representation of the database entity-relationship model.

Power Hardware In-the-Loop (PHIL) is a test system technique which combines the system simulation flexibility with the fidelity of complete hardware test. It is based on a Digital Real-Time Simulation (DRTS) that communicates with a Power Amplifier (PA), which exchanges real power with the Hardware-Under-Test (HUT). This data article describes a database which contains information about PHIL tests, organized in eight different interconnected dynamic tables. The Unified Modeling Language (UML) representation of these dynamic tables is shown in Fig. 1. The variables contained in every table and their description are shown in Table 1, Table 2, Table 3, Table 4, Table 5, Table 6, Table 7, Table 8.

**Table 1:** Variable description of “HUT Device” table.

HUT Device		
Variable	Type	Description
Id	Integer	Item Identifier
Device	Categorical	Name of the device
HUT_Types	Categorical	Type of Hardware Under Test (HUT), taken from the dynamic table “HUT Types”
Company-University	Categorical	Name of the company, university and/or research centre which develops the item, taken from the dynamic table “Companies_Universities”
Added_By	Categorical	User who adds the item
Add_Date	Date	Date of the item addition, in dd/mm/yyyy format
Revision_Date	Date	Date of the last revision of the item, in dd/mm/yyyy format
Permission_User	Categorical	Users who have the permission to modify the item
Last_Modification_Date	Date	Date of the last modification of the item, in dd/mm/yyyy format
Last_modification_By	Categorical	User who made the last modification

**Table 2:** Variable description of “HUT Types” table.

HUT Types		
Variable	Type	Description
Id	Integer	Item Identifier
HUT	Categorical	The different types of Hardware Under Test (HUT) used in the experiments: Circuit Breaker, Car: FTP-72 driving cycle, Nonlinear circuit, Linear Circuit, SFCL (Superconducting Fault Current Limiter), OCR (Overcurrent relays), Smart Transformer (ST), Battery Energy Storage System (BESS), Distributed Energy Storage Systems (DESS), PV Inverter, Virtual Synchronous Generator (VSG), High Speed Generator, AC/DC power conversion module (PCM), Voltage Source Converter (VSC), Electric Drive, Physical Analog Subsystem (PAS), Generator, Statcom, Wind Inverter, Battery Inverter and House. If it is not defined in the test, it is selected “not shown”

**Table 3:** Variable description of “Simulated System” table.

Simulated System		
Variable	Type	Description
Id	Integer	Item Identifier
Simulated System	Categorical	The different systems simulated in the Digital Real-Time Simulator (DRTS) in the experiments: Lithium Battery, Short-Circuit, Grid Voltage, Electric Ship, Electric Grid, PV, Wind Turbine, Electric Motor/Generator, Gas turbine generator, On Load Tap Changer (OLTC). If there is not a specific system, the field is empty

**Table 4:** Variable description of “Test Objective” table.

Test Objective		
Variable	Type	Description
Id	Integer	Item Identifier
Test Objective	Categorical	The main objective of the described test: Check PHIL Behaviour, Test HUT and/or Test Simulated System. If there is no main test objective specified, the field is empty

**Table 5:** Variable description of “Companies_Universities” table.

Companies Universities		
Variable	Type	Description
Id	Integer	Item Identifier
Company-University Name	Categorical	Full name of the company, university or research centre
Research_Center	Binary	1: yes, the item is a research centre
		0: no, the item is not a research centre
University	Binary	1: yes, the item is a university
		0: no, the item is not a university
Company	Binary	1: yes, the item is a company
		0: no, the item is not a company
Added_By	Categorical	User who adds the item
Add_Date	Date	Date of the item addition, in dd/mm/yyyy format
Revision_Date	Date	Date of the last revision of the item, in dd/mm/yyyy format
Permission_User	Categorical	Users who have the permission to modify the item
Last_Modification_Date	Date	Date of the last modification of the item, in dd/mm/yyyy format
Last_modification_By	Categorical	User who made the last modification

**Table 6:** Variable description of “Papers” table.

Papers		
Variable	Type	Description
Id	Integer	Item Identifier
Year	Integer	Year of publication
Title	Categorical	Name of the paper
Authors	Categorical	Authors of the paper
Company-University	Categorical	Companies, universities and/or research centres which carried out the test. These data are taken from the dynamic table “Companies-Universities” of Table 5
Step Time (μsec)	Numerical	Real-time simulation step-time in μsec
DRTS	Categorical	Digital Real-Time Simulator used in the experiment. These data are taken from the dynamic table “DRTS” of Table 8
Test Power (kVA)	Numerical	Maximum power achieved during the test in kVA
Interconnection Method	Categorical	Type/s of interconnection method between DRTS and PA: Analog signals, digital signals or not shown.
Algorithm	Categorical	Algorithm/s used during the test: Ideal Transformer Model (ITM), Transmission Line Model (TLM), Damping Impedance Method (DIM), Time-variant First-order Approximation (TFA), Partial Circuit Duplication (PCD)
Power Amplifier	Categorical	Power Amplifier used in the experiment. These data are taken from the dynamic table “Power Amplifier” of Table 7
HUT Type	Categorical	Type of Hardware Under Test (HUT) used in the experiment. These data are taken from the dynamic table “HUT Types” of Table 2
Simulated System	Categorical	System simulated in real-time in the Digital Real-Time Simulator (DRTS). These data are taken from the dynamic table “Simulated System” of Table 3
Test Objective	Categorical	Main goal of the experiment. These data are taken from the dynamic table “Test Objective” of Table 4
HUT Device	Categorical	Specific device used as a Hardware Under Test (HUT) in the experiment. These data are taken form the dynamic table “HUT Device” of Table 1
Added_By	Categorical	User who adds the item
Add_Date	Date	Date of the item addition, in dd/mm/yyyy format
Revision_Date	Date	Date of the last revision of the item, in dd/mm/yyyy format
Permission_User	Categorical	Users who have the permission to modify the item. Only the registered users can see this variable
Last_Modification_Date	Date	Date of the last modification of the item, in dd/mm/yyyy format
Last_modification_By	Categorical	User who made the last modification

**Table 7:** Variable description of “Power Amplifier” table.

Power Amplifier		
Variable	Type	Description
Id	Integer	Item Identifier
Model	Categorical	Model name of the Power Amplifier (PA). If the brand of the PA is specified but not the model, the name of the brand with the label “(no model)” has been included in the database
Power (kW)	Numerical	Maximum power of the PA in kW
Voltage BW (Hz)	Numerical	Maximum bandwidth of the output voltage in Hz
Current BW (Hz)	Numerical	Maximum bandwidth of the output current in Hz
Accuracy (pct)	Numerical	Output accuracy of the PA in %
Width (mm)	Numerical	Width of the PA enclosure in mm
Height (mm)	Numerical	Height of the PA enclosure in mm
Depth (mm)	Numerical	Depth if the PA enclosure in mm
Weight (kg)	Numerical	Weight of the PA in kg
Power density (kW_div_dm3)	Numerical	Power density of the PA in kW/dm3
Specific Power (kW_div_kg)	Numerical	Specific power of the PA in kW/kg
Voltage Range (V)	Numerical	Range of the output voltage of the PA in V
Current Range (A)	Numerical	Range of the output current of the PA in A
Efficiency (pct)	Numerical	Efficiency of the PA in %
Voltage Ripple (pct)	Numerical	Maximum ripple of the output voltage of the PA in %
Price (€)	Numerical	Price of the PA in €
Slew Rate (V_div_μsec)	Numerical	Slew rate if the PA in V/μsec
Delay (μsec)	Numerical	Delay between the input and output of the PA in μsec
Communication	Categorical	Type/s of communication with the DRTS: analog, digital and/or optical link
Quadrants	Integer	Quadrants in which the PA can operate: 1,2,3 and/or 4.
Modularity	Categorical	Degree of combination of several power amplifiers: serialize, parallelize or nothing.
Power Factor	Numerical	Power factor of the PA in cos φ
Portability	Categorical	Portability of the PA: rack format, wheels, forklift openings or nothing
Security	Categorical	Protections of the PA: overtemperature, overvoltage, overcurrent, emergency stop or not show
Standards	Categorical	Standards accomplished by the PA: IEC/EN 50178, IEC/EN 50581, IEC/EN 61000-2-2, IEC/EN 61000-4-4, IEC/EN 61000-4-5, IEC/EN 61000-4-8, IEC/EN 61000-4-11, IEC/EN 61000-4-13, IEC/EN 61000-4-14, IEC/EN 61000-4-17, IEC/EN 61000-4-27, IEC/EN 61000-4-28, IEC/EN 61000-4-29, IEC/EN 61000-4-34, IEC/EN 61000-6-2, IEC/EN 61000-6-4, IEC/EN 60146-1-1, IEC/EN 60529, IEC/EN 61131–2, IEC/EN 61496–1, IEC/EN 61800–3, IEC/EN 62040–2, SEMI F47-0706, VDE 0126/EN 50438
Company-University	Categorical	Companies, universities and/or research centres which develop the PA. These data are taken from the dynamic table “Companies-Universities” of Table 5
Added_By	Categorical	User who adds the item
Add_Date	Date	Date of the item addition, in dd/mm/yyyy format
Revision_Date	Date	Date of the last revision of the item, in dd/mm/yyyy format
Permission_User	Categorical	Users who have the permission to modify the item
Last_Modification_Date	Date	Date of the last modification of the item, in dd/mm/yyyy format
Last_modification_By	Categorical	User who made the last modification

**Table 8:** Variable description of “DRTS” table.

DRTS		
Variable	Type	Description
Id	Integer	Item Identifier
Model	Categorical	Model name of the Digital Real-Time Simulator (DRTS)
Company-University	Categorical	Companies, universities and/or research centres which develop the DRTS. These data are taken from the dynamic table “Companies-Universities” of Table 5
Hardware	Categorical	Hardware used to run the simulation: CPU, DSP, GPU and/or FPGA
Host OS	Categorical	Operative System of the host: windows or Linux
Target OS	Categorical	Operating System of the target: Linux based, QNX, Red Hat, VxWorks, QNX RTOS, Optimized Real-time kernel, FPGA
Application Software	Categorical	Software used to model and run the simulation: Matlab/Simulink, RSCAD, RT-Lab, Hypersim software suite, AdvantageDE
Communication, Protocols, Interfacing and I/O	Categorical	Supported communication, protocols, interfacing and I/Os of the DRTS: Gigabit Ethernet, Dolphin networking, IEC61850, C37.118, DNP3, Shared memory, Third party I/Os, Optical fiber, Fast back plane, Global bus hub, TCP/IP, Analog and digital I/O, PCI, PCIe. PXI, PMC, IEEE 1284C, Serial, UDP/IP, CAN, J1939, SFP
Application	Categorical	Type of application of the DRTS: Power systems, Real-time simulation of power electronics, Control and automotive systems, Multi-domain simulation, HIL testing, Specialization in avionics and maritime, Control system and/or Rapid prototyping
ADC bit	Integer	Number of bits of the DRTS ADC
ADC delay	Numerical	Delay of the DRTS ADC in μsec
Minimum Time Step (μsec)	Numerical	Minimum time step of the DRTS in μsec
Added_By	Categorical	User who adds the item
Add_Date	Date	Date of the item addition, in dd/mm/yyyy format
Revision_Date	Date	Date of the last revision of the item, in dd/mm/yyyy format
Permission_User	Categorical	Users who have the permission to modify the item
Last_Modification_Date	Date	Date of the last modification of the item, in dd/mm/yyyy format
Last_modification_By	Categorical	User who made the last modification

The database is implemented in Microsoft Access [2]. This software provides the possibility of dynamic classification of all available parameters of Power Hardware In-the-Loop (PHIL) tests, allowing the organization of the information without requiring programming skills. Furthermore, a user management system (Fig. 4) and a GUI interface (Fig. 5, Fig. 6, Fig. 7) are included to add, modify and update the online database. It also offers the possibility of showing information as a table or as a form, and users can copy and paste data to other database software to process them.

## 2 Experimental design, materials, and methods

A significant number of PHIL test reports have been collected from the scientific literature. The main data were extracted from these publications for reproducibility purposes. Some representative examples of these publications gathered in the database are [[3], [4], [5], [6], [7], [8]]. The information of PHIL systems used in the tests was completed by extracting data from manufacturer datasheets and from PHIL review articles [[9], [10], [11], [12], [13], [14], [15], [16]]. Publications with insufficient information were excluded.

**Fig. 2:**
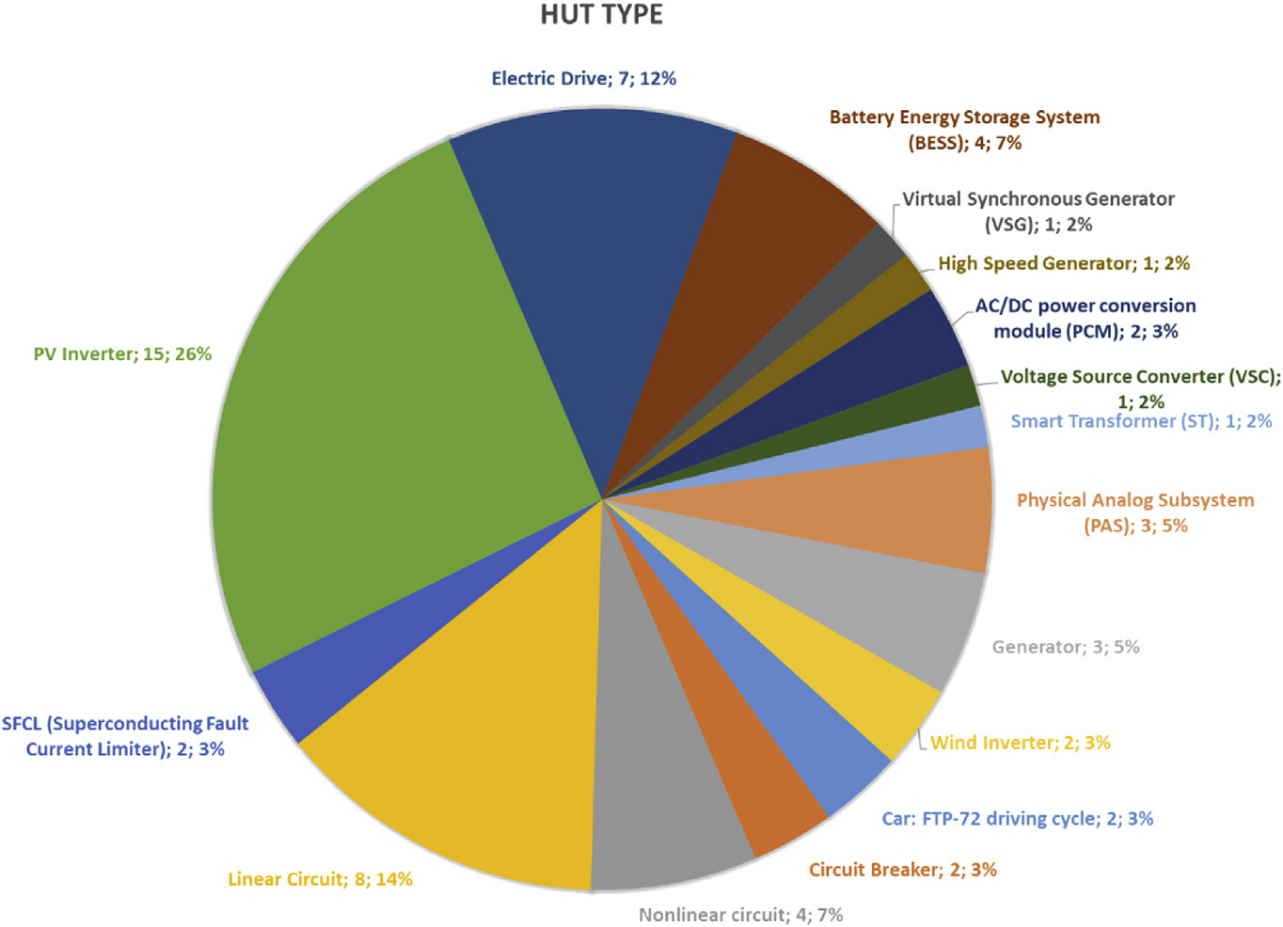
Percentage of the different HUT types used in the database tests.

**Fig. 3:**
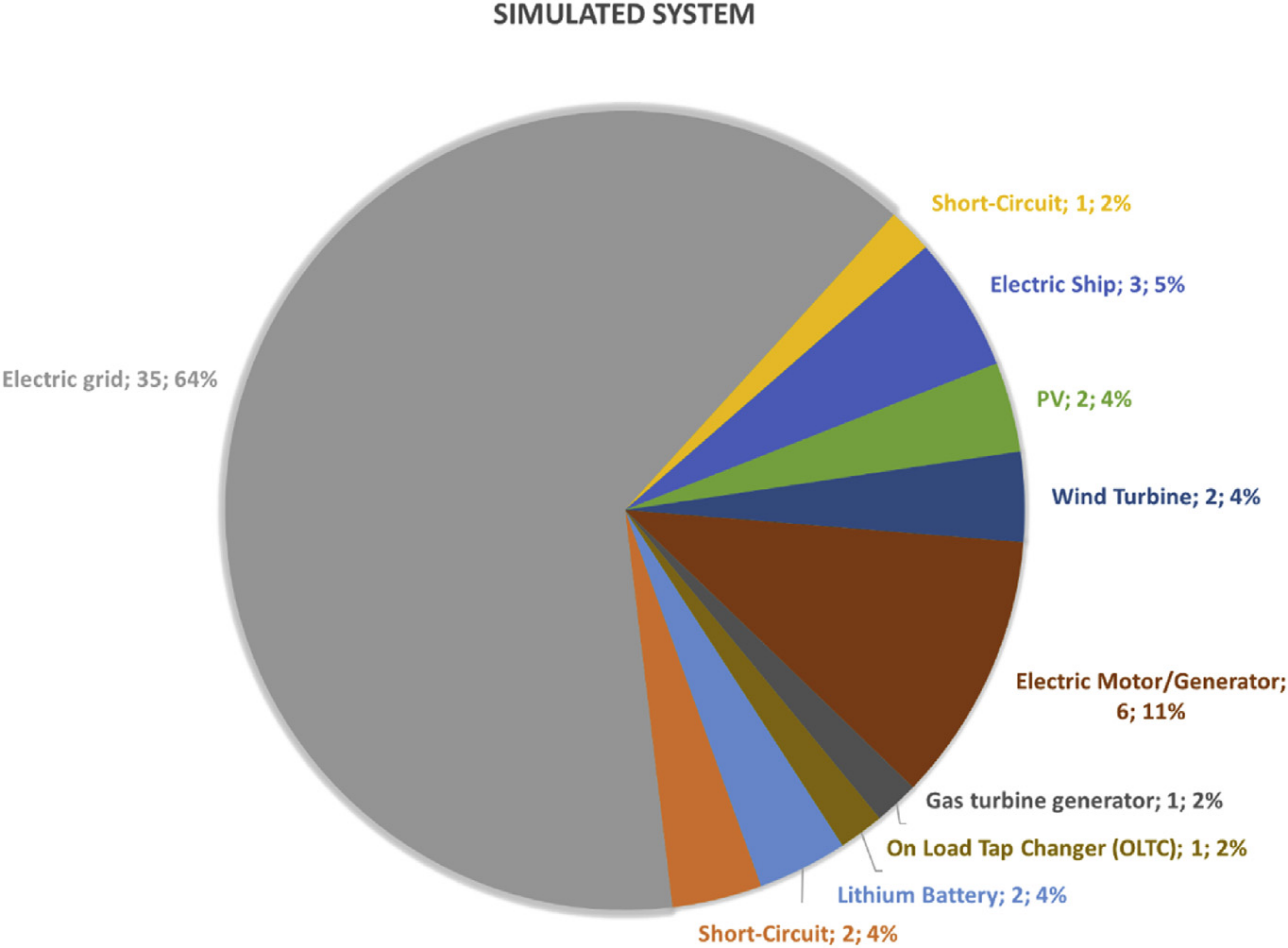
Percentage of the different systems simulated in real-time in the DRTS of the database tests.

Summary statistics of one of the most important variables included in the database are presented in Fig. 2, Fig. 3. If any paper describes two or more tests, in which the same HUT type has been test or the same system has been simulated [7,8], it only counts once.

In order to increase the comprehension and traceability of the items in the database, some non-categorizable fields have been added. A field called “Notes” has been added in every table, with the purpose of adding some extra information which is interesting for the readers. Furthermore, a web link of the item has been added in Table 5, Table 6, Table 7, Table 8. Moreover, the “Paper” table (Table 6) includes a “Summary”, “Why and what for”, “Results” and “Conclusions” to add the subjective information of the paper. The table “Power Amplifier” (Table 7) includes a field “attachment” where the datasheet of the system can be added.

Several methods have been considered in order to ensure information integrity, to increase readability, and to update the information periodically. These methods are described in the following subsections:

### 2.1 User management

**Fig. 4:**
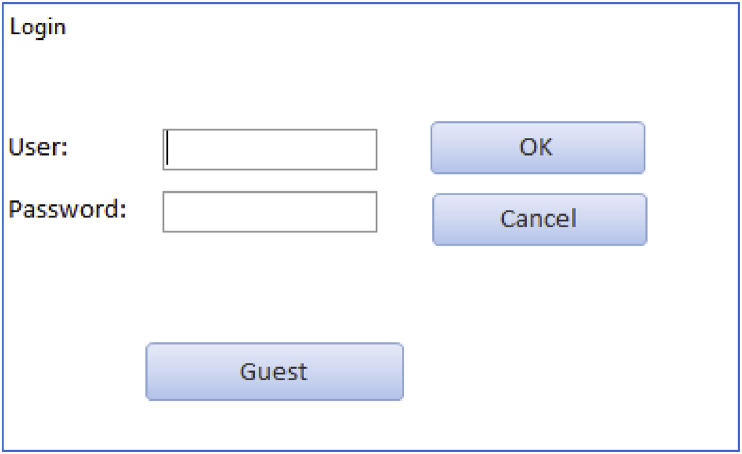
Login window of the database. The “Guest button” allows non-registered users to read the database.

The database allows modification after its publication. A user management system has been implemented to prevent anonymous database editing. The registration is done via email, with every user having a unique username and password to enter the application. Only registered users are able to add new information to the database. Furthermore, only users with special rights can modify the information. Consequently, the database records the date and authorship of every addition/modification. Finally, a “guest” user allows non-registered users to read the database. Fig. 4 shows the login window to enter to the online database.

**Fig. 5:**
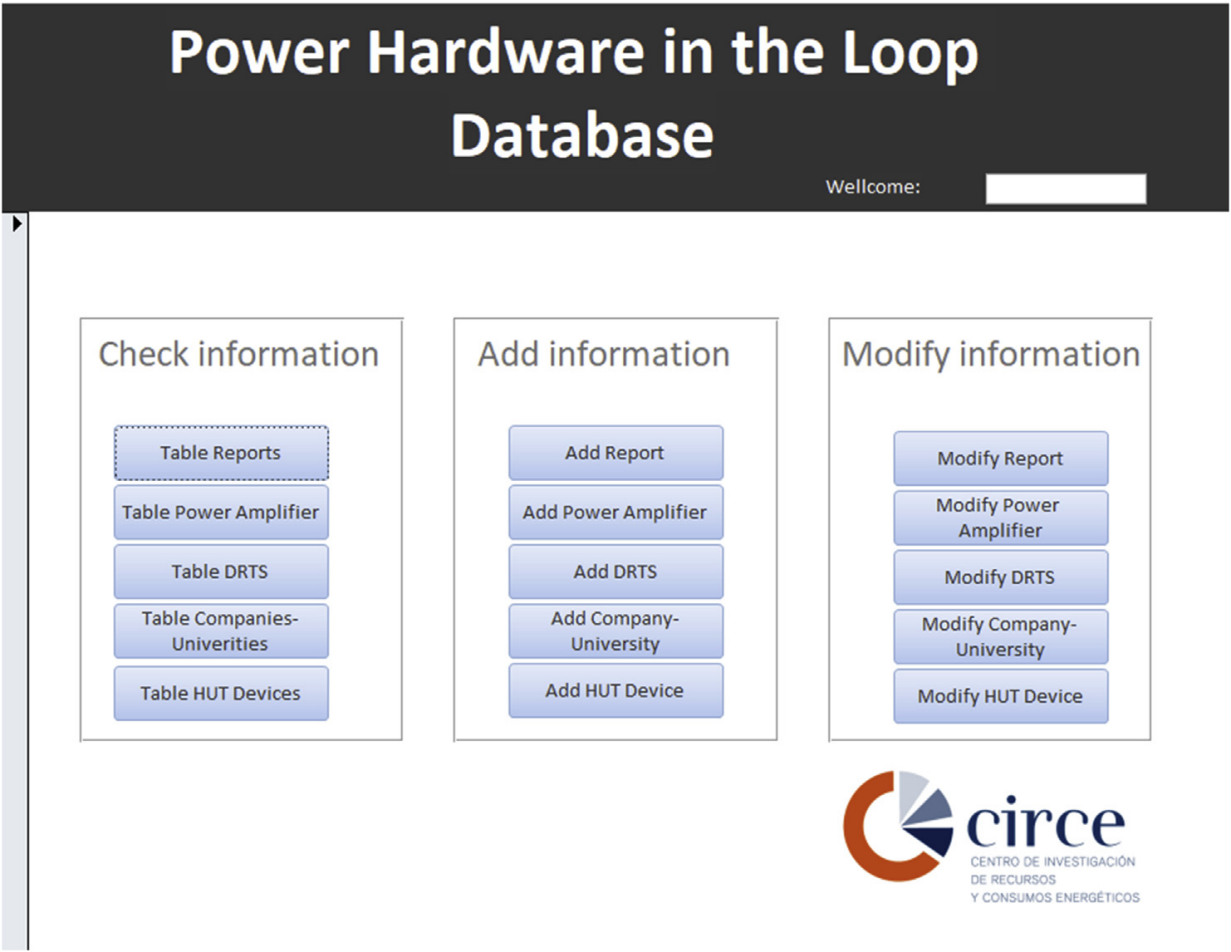
PHIL database user interface. Three menu bars are available: check, add and modify information.

**Fig. 6:**
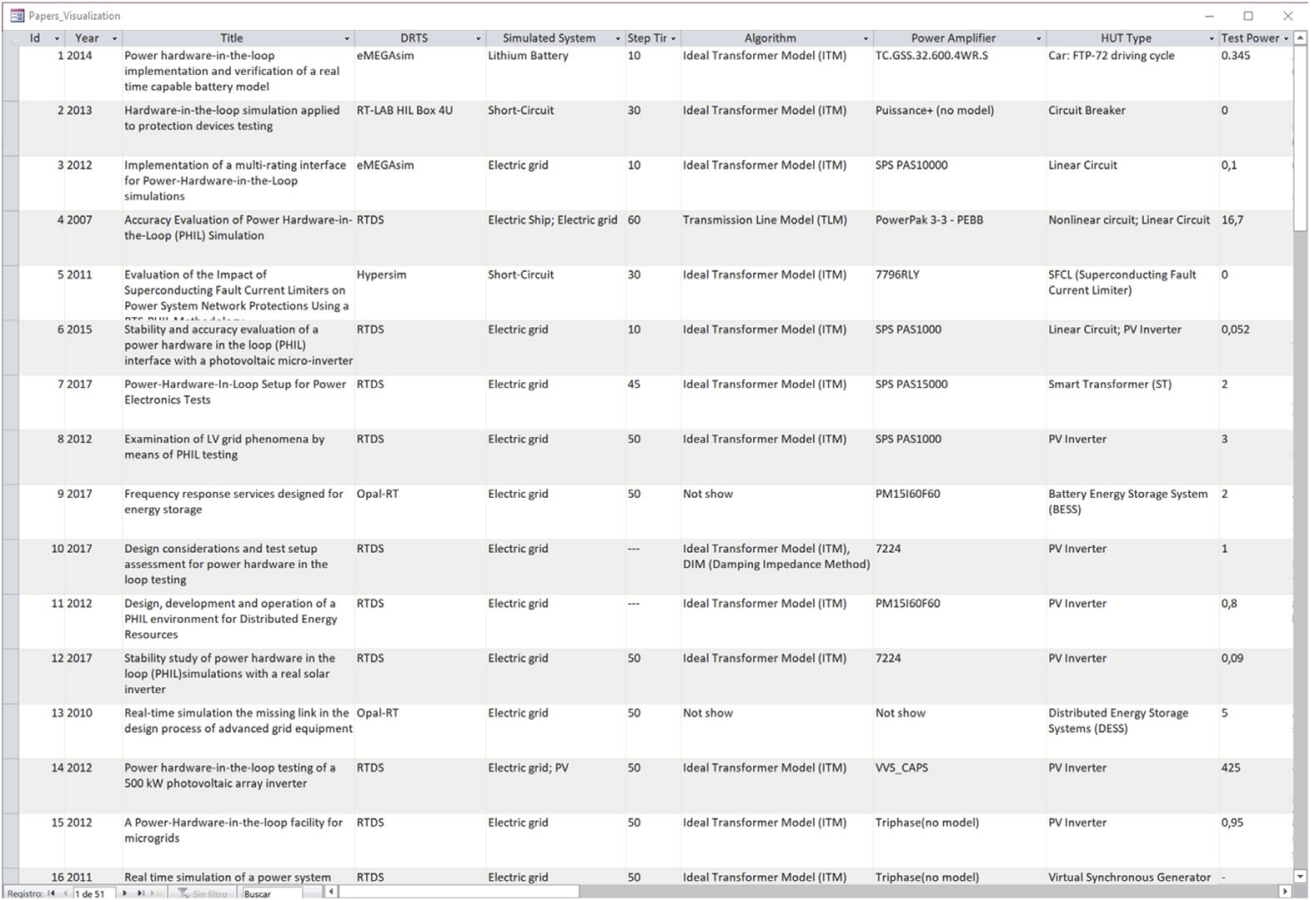
“Table Reports” in the database GUI (Fig. 5), which contains the PHIL test manuscripts information of the database.

**Fig. 7:**
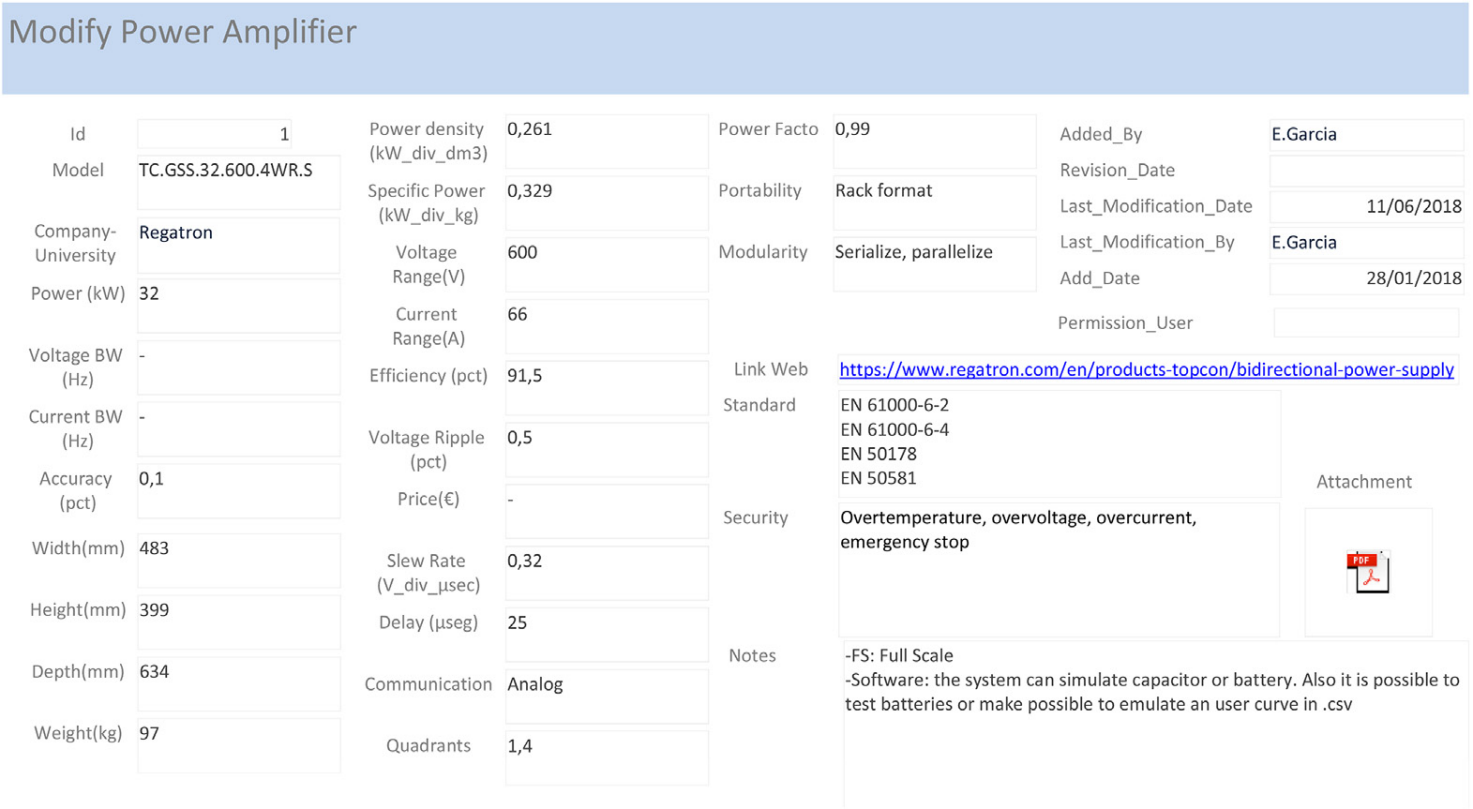
“Modify Power Amplifier” in the database GUI (Fig. 5), which gives access to the Power Amplifier data change form.

### 2.2 Database GUI

Fig. 5 shows the database GUI. It has three main groups: check, add and modify information. Each group allows users to check, add or modify the reports, Power Amplifiers (PA), Digital Real-Time Simulator (DRTS), Companies/Universities and Hardware-Under-Test (HUT) device tables respectively. All users can access the items in the check information group. However, to enter the other two groups it is necessary to be registered in the database.

Fig. 6 shows the PHIL tests report table which holds all the information included in the reviewed publications and it is accessed by the “Table Reports” button in the database GUI (Fig. 5). Fig. 7 shows the “form” to change PA data, which is opened by clicking the “Modify Power Amplifier” button in the database GUI (Fig. 5).

### 2.3 Database host

The database is uploaded to GitHub [1], which is an open-source version control system. It gives the possibility of changing and updating the database in an orderly manner, preventing the database from becoming outdated. However, since the Access database must be updated in GitHub as a binary file, only the main branch of the database could be useful to users. This form of centralised management enables better organised control of the database updating process. Consequently, this supervision technique will provide a third-party revision to prevent mistakes and any attempts of cheating.
